# Social Risk Burden among US Cancer Survivors across Adulthood: Evidence from the 2022–2023 BRFSS

**DOI:** 10.1158/2767-9764.CRC-25-0664

**Published:** 2026-03-16

**Authors:** Ami E. Sedani, Bijal A. Balasubramanian, Stephanie B. Wheeler, Anisha Ganguly

**Affiliations:** 1 Department of Epidemiology, UTHealth Houston School of Public Health, Dallas, Texas.; 2 UTHealth Houston Institute for Implementation Science, Houston, Texas.; 3 Department of Health Policy and Management, Gillings School of Global Public Health, University of North Carolina at Chapel Hill, Chapel Hill, North Carolina.; 4 Lineberger Comprehensive Cancer Center, University of North Carolina at Chapel Hill, Chapel Hill, North Carolina.; 5 Division of General Medicine and Clinical Epidemiology, School of Medicine, University of North Carolina at Chapel Hill, Chapel Hill, North Carolina.

## Abstract

Cancer can exacerbate social vulnerabilities, undermining follow-up care, screening, and survivorship. Yet, nationally representative data on multiple social risks among survivors are limited. This study estimated the prevalence of social risks among cancer survivors relative to adults without a cancer history. We analyzed 2022 to 2023 Behavioral Risk Factor Surveillance System data (*n* = 472,531; weighted *n* = 265.6 million). Seven social risks were assessed: food insecurity (two items), housing insecurity, utility insecurity, employment insecurity, transportation insecurity, and cost-related barrier to care. We estimated prevalence and absolute differences (AD) by cancer history, stratified by age group. Among young adults (18–39), analyses were further stratified by Medicaid expansion status, race and ethnicity, and sex. Overall, 8.7% of adults reported a history of cancer. Survivors had higher prevalence of social risks in young adulthood, attenuated differences in middle age (40–64 years), and absent or reversed differences in older adulthood (65+). Among young adult survivors, ADs were larger in non–Medicaid expansion states for Supplemental Nutrition Assistance Program participation, housing and utility insecurity, and cost-related barrier to care. ADs were generally larger among non-Hispanic White adults, although racially and ethnically minoritized groups experienced higher prevalence across all social risks. ADs were also larger among males than females. Young adult cancer survivors bear the highest burden of social risks, with notable differences by Medicaid expansion status and demographic factors such as race and ethnicity and sex.

**Significance:**

Age-stratified, population-based comparisons of social risks between cancer survivors and adults without a cancer history provide actionable baselines for survivorship care and policy design, particularly for younger survivors.

## Introduction

The growing population of individuals with a cancer history (“cancer survivors”) poses a significant public health challenge. As of 2022, more than 18 million individuals in the United States had a history of cancer, a number projected to exceed 26 million by 2040 (1). As survivors live longer, optimal outcomes depend not only on high-quality clinical care but also on addressing broader social and economic conditions that influence health and recovery across adulthood (2). These conditions, often referred to as social risks, include adverse social, economic, and environmental circumstances that affect health and healthcare access and are shaped by structural contexts (e.g., policy environments and institutional practices; ref. 3). Social risks intersect with cancer survivorship because treatment and recovery often disrupt employment, strain finances, and create lasting psychosocial challenges (4, 5).

Importantly, these challenges do not occur uniformly across the life course. Younger adults often face unstable employment and limited access to social safety nets, whereas older adults may experience fixed incomes and greater reliance on public programs. Such age-specific contexts can amplify or mitigate vulnerability to social risks after cancer, making age-stratified analyses critical for identifying disparities and tailoring interventions. Prior research has largely focused on medical financial hardship [material (e.g., problems paying medical bills), psychologic (e.g., worry about costs), and behavioral (e.g., delaying or forgoing care because of cost); refs. 6, 7], with more recent attention to other social risk domains such as food insecurity (8). However, the broader spectrum of social risks remains underexamined in population-based survivorship research, and little is known about how these risks differ across the life course (6). This gap is critical because developmental stages (from young adulthood through older age) are associated with distinct social and economic contexts, including differences in employment stability, family roles, and access to social safety nets. This gap is compounded by the absence of directly comparing social risks between cancer survivors and those without a history of cancer, leaving the extent to which cancer contributes to social risk burden unclear, and reliance on multivariable adjustment, which can obscure meaningful population-level differences and mask true disparities (9–15). These age-specific circumstances may interact with cancer survivorship in ways that amplify or mitigate vulnerability, yet few studies have directly compared social risks between cancer survivors and adults without a cancer history across age groups. Social risks are also consistently more prevalent among racially and ethnically minoritized populations and those with lower income, patterns that persist among cancer survivors. Taken together, these considerations underscore the need for age-stratified, population-based comparisons between survivors and adults without a cancer history.

In this study, we used 2022 to 2023 data from the Behavioral Risk Factor Surveillance System (BRFSS) to describe the prevalence of seven individual-level social risks among cancer survivors compared with adults without a cancer history. We assessed differences overall and stratified by age group [18–39 years (young adults), 40–64 years, and ≥65 years] to reflect the heterogenous survivorship experiences across the life course. Given recent reductions in social safety net supports (16) and growing evidence of inequities among younger survivors, we also conducted exploratory analyses among young adults to examine variation by state Medicaid expansion status and across demographic groups (race and ethnicity and sex). These descriptive findings are intended to provide a timely, nationally representative snapshot of social risks in survivorship and inform clinical screening practices, survivorship care planning, and policy strategies to promote equitable outcomes.

## Materials and Methods

### Study design and data source

This cross-sectional descriptive study used publicly available, deidentified data from the 2022 and 2023 BRFSS; therefore, institutional review board approval was not required. This study follows the Strengthening the Reporting of Observational Studies in Epidemiology guideline for cross-sectional studies (17), the American Association for Public Opinion Research guideline for survey studies (18), and the descriptive epidemiology framework proposed by Lesko and colleagues (19).

The BRFSS is a nationally representative, cross-sectional telephone survey conducted annually among noninstitutionalized US adults (≥18 years of age; ref. 20). It is a collaborative effort between all 50 states, the District of Columbia (DC), and participating US territories and the Centers for Disease Control and Prevention (CDC; ref. 20). The BRFSS questionnaire consists of (i) a mandatory core component (e.g., health status and healthcare access) administered by all participating states, (ii) optional modules that states may choose to include [e.g., social determinants and health equity (SDHE)], and (iii) state-added questions tailored to local public health priorities (20). Further details on methodology are available from the CDC and in Supplementary Methods S1.

### Study sample

To create the analytic sample, we restricted the 2022 and 2023 BRFSS dataset to respondents residing in states or jurisdictions that administered the SDHE optional module, excluding the two participating US territories (Supplementary Fig. S1). Data from both years were pooled to enhance statistical precision and improve subgroup analyses, particularly for smaller subpopulations. Participants with missing demographic information from the core modules (“do not know/not sure,” refused, or missing) were excluded. Two exceptions were made—for annual household income [used for federal poverty level (FPL)] and primary source of health insurance, and “missing” categories were retained due to concerns about nonrandom missingness. Lastly, participants with missing data for any of the social risk items (*n* = 83,938; 13%) were excluded from the analytic sample. Supplementary Figure S2 shows states that administered the SDHE module in 2022, 2023, and the combined analytic sample (46 states plus DC).

### Measures

Cancer history was determined based on the core module item asking whether respondents had ever been told by a doctor, nurse, or other health professional that they had melanoma or any other type of cancer. Individuals with a history of nonmelanoma skin cancer were classified among those without a cancer history.

We included seven individual-level social risks: six from the SDHE optional module [employment stability, food security (two items), housing security, utility security, and transportation access], as well as one item from the core module (cost-related barrier to care). A summary measure of the number of social risks was created. Full questions, response categories, and coding decisions are provided in Supplementary Table S1. Consistent with prior conceptual work, participation in the Supplemental Nutrition Assistance Program (SNAP) is interpreted here as a proxy for underlying financial strain and structural vulnerability, rather than as an indicator of program inadequacy or failure.

Sociodemographic and contextual variables for descriptive and stratified analyses included (biological) sex, age, race and ethnicity, educational attainment, employment status, marital status, primary source of health insurance coverage, FPL, home ownership, urban–rural status, US census region, state Medicaid expansion status, veteran status, functional disability status, general health status, cigarette smoking status, having a personal healthcare provider, and time since last routine check-up. Race and ethnicity were self-reported using the combined BRFSS variable, consistent with guidance to minimize misclassification of Hispanic/Latino individuals (21). Categories were used as proxies for exposure to structural racism, reflecting social and political determinants of health relative to non-Hispanic (NH) White adults (21). Annual household income was converted to a continuous measure using midpoints of the eight BRFSS income categories and then used to calculate percent FPL based on household size and state-specific thresholds (22). Medicaid expansion status for each respondent’s state of residence was obtained from the Kaiser Family Foundation for the corresponding survey year (2022 or 2023; ref. 23).

### Analytic approach

Data were obtained in January 2025 and analyzed in SAS 9.4 (SAS Institute) from February to May 2025. Sampling weights were applied per CDC guidelines to produce population-representative prevalence estimates (https://www.cdc.gov/brfss/index.html). Data cleaning procedures were applied to define the final analytic sample. Given the descriptive focus, we report unadjusted prevalence estimates to align with guidance emphasizing the value of raw distributions for public health decision-making and avoid obscuring disparities through adjustment (19, 24–26).

We first stratified all estimates by age group: 18 to 39 years, 40 to 64 years, and ≥65 years. Among young adults, social risks were further stratified by Medicaid expansion status, race and ethnicity, and sex to enable a more nuanced understanding of disparities in social risks. We report counts and weighted percentages, as well as prevalence estimates with corresponding 95% confidence intervals (CI). Prevalence estimates and their 95% CIs were calculated using Taylor series linearization methods via PROC SURVEYFREQ, which account for the complex survey design and weighting structure of the BRFSS dataset. Absolute differences (AD) in social risk prevalence between cancer survivors and those without a history of cancer were calculated to quantify observed disparities, along with their 95% CIs. AD represents the unadjusted difference in prevalence (in percentage points) between cancer survivors and nonsurvivors within each stratum. Standard errors for ADs were derived by pooling the standard errors from each group, and 95% CIs were computed using the normal approximation.

### Sensitivity analysis

To assess potential bias from differential nonresponse to the SDHE module, we compared the characteristics of excluded participants (Supplementary Table S2). We also further investigated patterns of missing income data as household income is a key determinant of social risk and was used to calculate FPL (Supplementary Table S3). Based on the initial descriptive results, which showed increasing prevalence of missing income with age group, we assessed whether missingness was systematic by examining its distribution across age groups, cancer history, and selected social risks (SNAP participation and cost-related barriers to care).

## Results

### Sample characteristics

An unweighted sample of 472,531 individuals (representing a weighted sample of 265,643,293) was analyzed (Supplementary Table S4). Overall, 52.5% were female, 61.9% were NH White, 50.5% had commercial health insurance coverage, and 93.1% lived in urban areas.

A total of 58,077 participants (8.7%) self-reported a history of cancer, including 1.5% of young adults, 7.4% of adults ages 40 to 64 years, and 20.9% of those 65 years and older. Across all age groups, there was increased representation among individuals with a history of cancer identifying as NH White, college-educated individuals, homeowners, veterans, those with functional disability, poor baseline health, and former cigarette use; a majority of individuals reported having a personal healthcare provider and a routine check-up in the past year (Table 1).

**Table 1.** Characteristics of study sample according to self-reported cancer history, by age group (*n* = 472,531).

Characteristic	*n* (wt. %)					
	18–39 years [*n* = 100,628 (34.3%)]		40–64 years [*n* = 188,440 (41.3%)]		65+ years [*n* = 183,463 (24.4%)]	
	Cancer survivors	No history of cancer	Cancer survivors	No history of cancer	Cancer survivors	No history of cancer
Total	1,689 (1.5)	98,939 (98.5)	16,063 (7.4)	172,377 (92.6)	40,325 (20.9)	143,138 (79.1)
Sex	​	​	​	​	​	​
Male	588 (37)	50,634 (49.4)	5,767 (36.1)	80,923 (48.7)	18,908 (47)	61,338 (43.8)
Female	1,101 (63)	48,305 (50.6)	10,296 (63.9)	91,454 (51.3)	21,417 (53)	81,800 (56.2)
Age group, years	​	​	​	​	​	​
18–24	173 (14.7)	24,660 (31.7)	NA	NA	NA	NA
25–34	700 (45.1)	45,783 (46)	NA	NA	NA	NA
35–44	816 (40.2)	28,496 (22.3)	1,173 (9.4)	30,249 (22.3)	NA	NA
45–54	NA	NA	4,347 (30.1)	63,512 (37.9)	NA	NA
55–64	NA	NA	10,543 (60.5)	78,616 (39.9)	NA	NA
65+	NA	NA	NA	NA	40,325 (100)	143,138 (100)
Race and ethnicity	​	​	​	​	​	​
NH American Indian or Alaska Native	Suppressed*	1,775 (1.1)	256 (1.8)	3,106 (1.3)	309 (0.8)	1,550 (1)
NH Asian or Pacific Islander	Suppressed*	5,283 (8)	178 (3.5)	4,495 (6.3)	225 (1.9)	1,907 (3.5)
NH Black	73 (6.5)	8,184 (11.9)	801 (7.6)	14,842 (12.7)	1,563 (6.1)	9,707 (10.5)
NH multiracial	89 (5.6)	3,747 (4.2)	385 (3.3)	4,158 (3.2)	556 (2.5)	2,262 (2.3)
NH White	1,259 (63.3)	63,611 (51.9)	13,707 (75.1)	130,966 (60.1)	36,981 (84.8)	123,079 (74.8)
Hispanic	190 (17)	16,339 (22.9)	736 (8.7)	14,810 (16.3)	691 (3.9)	4,633 (7.9)
Educational attainment	​	​	​	​	​	​
Did not graduate HS	95 (8)	5,609 (10.5)	663 (7.7)	9,205 (10.9)	1,435 (7.1)	6,665 (10.7)
HS diploma/GED	381 (27.3)	26,814 (30.8)	3,250 (21.7)	36,797 (22.9)	8,884 (24.7)	34,940 (27)
Some college	524 (33.6)	26,616 (30.9)	4,624 (33.5)	45,479 (30.2)	11,171 (33.8)	40,047 (31.5)
College graduate^a^	689 (31.1)	39,900 (27.9)	7,526 (37)	80,896 (36)	18,835 (34.4)	61,486 (30.8)
Employment status	​	​	​	​	​	​
Working	1,241 (69.6)	75,773 (71.5)	9,502 (58.9)	125,058 (71.8)	5,466 (13.9)	24,804 (17.5)
Out of work, looking	116 (7.9)	6,281 (7.5)	727 (4.6)	7,609 (4.9)	345 (0.9)	1,486 (1.2)
Homemaker or student	179 (12.1)	14,197 (17.8)	638 (4.7)	8,363 (5.9)	993 (2.7)	4,118 (3.4)
Retired	Suppressed*	181 (0.2)	2,491 (15.3)	15,960 (8.4)	32,180 (78)	107,759 (73.2)
Unable to work	139 (9.6)	2,507 (2.9)	2,705 (16.6)	15,387 (9.1)	1,341 (4.5)	4,971 (4.6)
Marital/partnership status	​	​	​	​	​	​
Married	754 (42.4)	36,902 (33.5)	9,804 (63.4)	107,502 (64.1)	21,982 (57.9)	76,417 (57)
Divorced/separated/widowed	220 (12.4)	6,852 (6.2)	4,177 (23.6)	38,532 (20.1)	15,789 (36.6)	55,996 (35.9)
Never married	715 (45.2)	55,185 (60.3)	2,082 (13)	26,343 (15.8)	2,554 (5.5)	10,725 (7.1)
Insurance type	​	​	​	​	​	​
Commercial (private)	969 (54.8)	61,204 (56.6)	10,182 (63.8)	116,981 (66.4)	4,972 (13.5)	20,278 (15.2)
Public	​	​	​	​	​	​
Medicare	88 (7.2)	3,502 (4.7)	2,078 (12.3)	12,749 (7.5)	31,517 (76.5)	108,130 (72.9)
Medicaid	380 (22.9)	14,701 (16.3)	2,004 (12.3)	18,781 (10.9)	1,071 (2.9)	4,800 (4.3)
Other public/government	98 (5)	5,824 (6.2)	1,201 (7.1)	11,649 (6.9)	2,223 (5.4)	7,098 (5)
Uninsured	121 (7.1)	9,834 (11.6)	427 (3.3)	10,053 (6.9)	92 (0.3)	840 (0.8)
Do not know/not sure	Suppressed*	3,874 (5.2)	171 (1.1)	2,164 (1.4)	450 (1.5)	1,992 (1.7)
FPL	​	​	​	​	​	​
Under 138%	269 (30.9)	11,599 (23.3)	714 (15.4)	9,802 (15.8)	322 (4.6)	1,939 (8.2)
139%–250%	204 (17.9)	9,899 (16.5)	590 (10.7)	9,659 (12.6)	351 (5.2)	1,405 (4.6)
251%–400%	185 (14.5)	9,881 (15)	765 (13.1)	13,267 (16.8)	231 (2.6)	983 (3.2)
>400%	216 (19.1)	9,973 (15.4)	1,151 (22.3)	20,494 (27.3)	164 (2.1)	627 (2.2)
Missing^b^	168 (17.7)	14,459 (29.8)	2,006 (38.5)	19,985 (27.5)	7,927 (85.4)	28,104 (81.7)
Home ownership	​	​	​	​	​	​
Own	885 (57.9)	42,628 (47.2)	12,803 (83.9)	133,168 (79.8)	35,323 (90.7)	123,120 (88.2)
Rent	679 (34.3)	44,920 (39.8)	2,816 (14)	34,784 (18)	4,151 (7.7)	16,768 (9.8)
Other arrangement	125 (7.8)	11,391 (13.1)	444 (2.1)	4,425 (2.2)	851 (1.6)	3,250 (2)
Area of residence	​	​	​	​	​	​
Urban	1,474 (93.7)	88,824 (94.3)	13,852 (92.3)	149,031 (93.2)	34,197 (91.8)	119,862 (91.4)
Rural	215 (6.3)	10,115 (5.7)	2,211 (7.7)	23,346 (6.8)	6,128 (8.2)	23,276 (8.6)
US census region	​	​	​	​	​	​
Northeast	294 (13.8)	18,445 (14.6)	3,324 (14.8)	34,665 (14.9)	8,248 (15)	28,581 (14.8)
Midwest	501 (27.8)	29,380 (26)	4,610 (26.3)	50,593 (25.2)	11,002 (25.7)	41,297 (26.4)
South	425 (31.6)	24,248 (33.4)	4,322 (36.4)	44,792 (34.7)	10,909 (34)	38,177 (34.9)
West	469 (26.7)	26,866 (25.9)	3,807 (22.5)	42,327 (25.2)	10,166 (25.3)	35,083 (23.9)
Medicaid expansion state	​	​	​	​	​	​
Yes	1,344 (75.6)	79,222 (74.3)	12,630 (72.1)	135,678 (73)	31,476 (73.9)	110,708 (72.7)
No	345 (24.4)	19,717 (25.7)	3,433 (27.9)	36,699 (27)	8,849 (26.1)	32,430 (27.3)
Veteran status	​	​	​	​	​	​
Yes	127 (8.6)	5,540 (5.1)	1,487 (9.2)	16,149 (8.7)	8,906 (21.7)	24,595 (16.3)
No	1,562 (91.4)	93,399 (94.9)	14,576 (90.8)	156,228 (91.3)	31,419 (78.3)	118,543 (83.7)
Functional disability status	​	​	​	​	​	​
Yes	626 (40)	21,829 (23.5)	5,669 (36)	43,066 (25.4)	18,462 (47)	55,487 (40)
No	1,063 (60)	77,110 (76.5)	10,394 (64)	129,311 (74.6)	21,863 (53)	87,651 (60)
General health status	​	​	​	​	​	​
Good or better health	1,202 (69.4)	87,533 (87.5)	11,245 (69.3)	143,493 (81.8)	29,407 (70.6)	115,855 (78.1)
Fair or poor health	487 (30.6)	11,406 (12.5)	4,818 (30.7)	28,884 (18.2)	10,918 (29.4)	27,283 (21.9)
Cigarette smoking status^c^	​	​	​	​	​	​
Current cigarette use	315 (21.6)	10,948 (10.8)	2,510 (15.7)	25,376 (14.6)	2,823 (7.8)	11,295 (8.5)
Former cigarette use	371 (18.5)	16,000 (14.7)	4,791 (29.4)	44,260 (25.2)	16,562 (41.4)	51,866 (36.1)
Never used cigarettes	1,003 (59.9)	71,991 (74.5)	8,762 (54.9)	102,741 (60.2)	20,940 (50.9)	79,977 (55.4)
Has personal provider	​	​	​	​	​	​
Yes	1,444 (85.8)	73,305 (72.8)	15,283 (94.8)	153,371 (88.1)	39,329 (97.9)	137,229 (95.8)
No	245 (14.2)	25,634 (27.2)	780 (5.2)	19,006 (11.9)	996 (2.1)	5,909 (4.2)
Time since check-up	​	​	​	​	​	​
1 year or less	1,254 (72.8)	64,495 (65.7)	14,103 (87.5)	136,798 (78.9)	37,825 (94)	130,567 (91.7)
More than 1 year	435 (27.2)	34,444 (34.3)	1,960 (12.5)	35,579 (21.1)	2,500 (6)	12,571 (8.3)
Survey year	​	​	​	​	​	​
2022	853 (51.6)	51,147 (50.3)	8,519 (51.9)	90,281 (50.5)	20,329 (50)	71,975 (49.9)
2023	836 (48.4)	47,792 (49.7)	7,544 (48.1)	82,096 (49.5)	19,996 (50)	71,163 (50.1)

Note: Percentages represent weighted column percentages. Percentages may not add to 100% due to rounding.

Abbreviations: GED, general education diploma; HS, high school; NA, not applicable; Wt, weighted.

Suppressed* = In alignment with BRFSS data suppression guidelines, categories were suppressed when the number of cases is less than 50 or SE is greater than 30% to reduce the likelihood of a breach of confidentiality; suppressed values were not excluded from percentages.

a College includes technical school.

b Missing = do not know/not sure/missing/invalid response.

c Defined based on lifetime cigarette use and current use; does not include e-cigarettes, cigars, or other tobacco products.

Within the young adult age group, cancer survivors as compared with those without cancer had a higher prevalence of being female, married, from a rural area, and report current cigarette smoking. They were also more likely to be insured by Medicaid, unable to work, have a household income ≤250% below FPL, and lack homeownership—indicators of socioeconomic disadvantage not observed to the same extent in older age groups. Similar patterns were observed among middle-aged adults (40–64 years), though less pronounced. On the other hand, in the 65 years and above age group, cancer survivors were more likely to be male and receive Medicare coverage as compared with those without a history of cancer.

### Social risks by age group

Across all respondents, food insecurity was the most reported social risk, with differences by age group (Supplementary Table S5). Overall, 25.6% of cancer survivors (one social risk, 13.1%; two social risks, 5%; and three or more social risks, 7.5%) and 35.4% of adults without a cancer history (one social risk, 16.9%; two social risks, 7.7%; and three or more social risks, 10.8%) reported one or more social risk factors (Supplementary Table S6). The prevalence of social risks generally decreased with age, regardless of cancer history.

Table 2 presents the prevalence of each social risk factor by cancer history and the corresponding AD, stratified by age group. The greatest ADs in social risks by cancer history were observed among young adults (18–39 years), although estimates in this subgroup were less precise (wider 95% CIs) due to smaller sample sizes.

**Table 2.** Prevalence of individual social risks and ADs among cancer survivors and adults without a cancer history, stratified by age group (*n* = 472,531).

Social risks	18–39 years			40–64 years			65+ years		
	Cancer survivors	No history of cancer	AD in Wt. prevalence, 95% CI	Cancer survivors	No history of cancer	AD in Wt. prevalence, 95% CI	Cancer survivors	No history of cancer	AD in Wt. prevalence, 95% CI
	Wt. prevalence (%), 95% CI	Wt. prevalence (%), 95% CI		Wt. prevalence (%), 95% CI	Wt. prevalence (%), 95% CI		Wt. prevalence (%), 95% CI	Wt. prevalence (%), 95% CI	
SNAP participation	22.3 (18.3–26.3)	13.5 (13.1–13.9)	8.8 (4.8–12.9)	12.7 (11.5–14)	11.1 (10.8–11.4)	1.7 (0.4–2.9)	5.4 (4.9–6)	7.1 (6.8–7.5)	−1.7 (−2.4 to −1.1)
Food insecurity	23.8 (19.6–28.1)	16.6 (16.2–17)	7.2 (3–11.5)	14.7 (13.4–16)	13.8 (13.4–14.1)	1 (−0.4 to 2.3)	7.5 (6.8–8.1)	8.9 (8.5–9.3)	−1.5 (−2.2 to −0.7)
Housing insecurity	24.6 (20.5–28.8)	15.3 (14.9–15.7)	9.4 (5.2–13.5)	13.1 (11.8–14.3)	12 (11.7–12.3)	1 (−0.2 to 2.3)	3.4 (2.9–3.8)	4.7 (4.3–5)	−1.5 (−1.8 to −0.7)
Utility insecurity	16.2 (12.3–20.1)	8.9 (8.6–9.2)	7.3 (3.4–11.2)	9.1 (8.1–10.1)	8.3 (8–8.6)	0.8 (−0.2 to 1.8)	2.4 (2–2.9)	3 (2.8–3.2)	−0.6 (−1 to −0.1)
Employment insecurity	23 (18.7–27.2)	19 (18.6–19.5)	3.9 (−0.4 to 8.3)	12.2 (11–13.5)	11.9 (11.5–12.2)	0.4 (−0.9 to 1.7)	2.6 (2.2–3)	3.2 (3–3.5)	−0.6 (−1.1 to −0.2)
Cost-related barrier to care	20.5 (16.6–24.4)	15.1 (14.7–15.5)	5.4 (1.5–9.3)	10.9 (9.8–12.1)	10.1 (9.8–10.4)	0.8 (−0.4 to 2)	2.5 (2.2–2.9)	3 (2.8–3.2)	−0.5 (−0.9 to 0)
Transportation insecurity	16.7 (12.7–20.7)	10.7 (10.3–11)	6.1 (2.1–10.1)	8.7 (7.5–9.8)	6.7 (6.5–6.9)	2 (0.8–3.1)	3.9 (3.4–4.4)	3.9 (3.7–4.2)	0 (−0.6 to 0.6)
Cumulative social risk, Mean (±STD)	1.47 (0.11)	0.99 (0.01)	0.48 (0.47–0.50)	0.81 (0.03)	0.74 (0.01)	0.08 (0.07–0.08)	0.28 (0.01)	0.34 (0.01)	−0.06 (−0.06 to −0.06)

Note: AD (survivor – no history). Percentages are weighted. The values in parenthesis following percentages are the corresponding 95% CIs of the estimate. Cumulative social risk ranged from 0–7.

Abbreviations: STD, standard deviation; Wt., weighted.

Among young adults, cancer survivors consistently reported a higher prevalence of social risks compared with their peers without a cancer history. Young adult survivors were more likely to report housing insecurity (AD: 9.4; 95% CI, 5.2–13.5), SNAP participation (AD: 8.8; 95% CI, 4.8–12.9), utility insecurity (AD: 7.3% points; 95% CI, 3.4–11.2), food insecurity (AD: 7.2; 95% CI, 3–11.5), and transportation insecurity (AD: 6.1; 95% CI, 2.1–10.1). There were additional differences observed in cost-related barrier to care (AD, 5.4; 95% CI, 1.5–9.3).

Among adults ages 40 to 64 years, differences in social risks by cancer history were generally minimal, whereas among those ages ≥65 years, cancer survivors often reported equal or lower prevalence of social risks compared with peers without cancer.

### Social risk variation among young adults

To better understand heterogeneity in social risks within young adults, we examined differences by state Medicaid expansion status, race and ethnicity, and sex.

#### Medicaid expansion status

**Figure 1.**
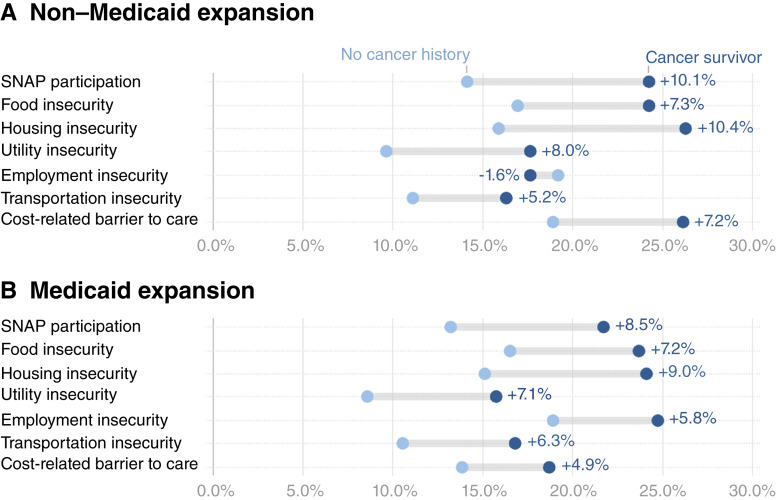
ADs in weighted prevalence (%) of individual social risks and 95% CIs for cancer survivors versus adults without a cancer history, stratified by Medicaid expansion status among young adults. ADs (cancer survivors minus adults without a cancer history) in prevalence of social risk factors among young adults (ages 18–39), stratified by state Medicaid expansion status. **A,** Non–Medicaid expansion states. **B,** Medicaid expansion states. Social risk factors include food insecurity (two items), housing insecurity, utility insecurity, employment insecurity, transportation insecurity, and cost-related barriers to healthcare. Estimates are weighted and derived from the 2022–2023 BRFSS.

Young adult cancer survivors reported higher prevalence of most social risks compared with those without a cancer history; however, employment insecurity in non–Medicaid expansion states was an exception (Fig. 1). ADs between young adults with and without a history of cancer were larger in non–Medicaid expansion states for SNAP participation, housing insecurity, utility insecurity, and cost-related barrier to care, whereas Medicaid expansion states showed larger ADs for employment and transportation insecurity. Overall, the prevalence of social risk factors was higher in nonexpansion states for adults with and without a history of cancer, with the exception of employment and transportation security among cancer survivors in Medicaid expansion states.

To complement these policy-level findings, we conducted a *post hoc* exploratory analysis stratified by insurance type to examine individual-level access patterns (Supplementary Fig. S3). Findings were generally consistent with expected gradients. Young adults with public insurance had the highest prevalence of social risks, regardless of cancer history, as well as the largest AD for all social risks, whereas those with private insurance had lower prevalence and smaller gaps. Sample sizes were insufficient to report estimates for young adults without health insurance (uninsured), except for housing and employment insecurity. Among these two social risks, uninsured young adults exhibited the highest prevalence for both those with and without a history of cancer and the largest ADs, compared with public and private insurance groups. Interpretation remains limited due to small cell sizes and BRFSS suppression guidelines (<50 unweighted), particularly for other uninsured categories and participants with missing insurance data.

#### Race and ethnicity

**Figure 2.**
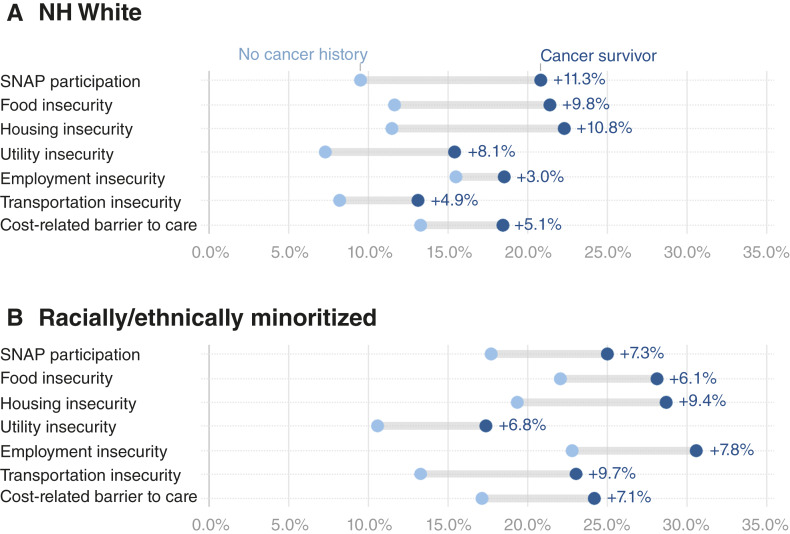
ADs in weighted prevalence (%) of individual social risks and 95% CIs for cancer survivors versus adults without a cancer history, stratified by race and ethnicity among young adults. ADs (cancer survivors minus adults without a cancer history) in prevalence of social risk factors among young adults (ages 18–39), stratified by race and ethnicity. **A,** NH White group. **B,** Racially/ethnically minoritized group. Social risk factors include food insecurity (two items), housing insecurity, utility insecurity, employment insecurity, transportation insecurity, and cost-related barriers to healthcare. Estimates are weighted and derived from the 2022–2023 BRFSS.

ADs between young adults with and without a history of cancer were larger among NH White individuals compared with corresponding differences among racially and ethnically minoritized groups, except for employment and transportation insecurity and cost-related barrier to care (Fig. 2). The larger ADs observed among NH White young adults for some social risk factors reflect the lower prevalence in this group. For example, 22% of racial and ethnic minority young adults without a cancer history reported food insecurity, compared with 11.6% of NH White young adults. Among cancer survivors, this prevalence was 28.1% and 21.4%, respectively. As a result, the AD by cancer history was 6.1% points among racially and ethnically minoritized groups, whereas it was 9.8 among NH White young adults. Compared with NH White young adults, the prevalence was higher for all seven social risks among racially and ethnically minoritized groups (regardless of cancer history). The prevalence of food (28.1%), housing (28.7%), and employment insecurity (30.6%) among racially and ethnically minoritized cancer survivors was higher than those observed in NH White survivors as well as than in other subgroups, including by Medicaid expansion status and sex.

#### Sex

**Figure 3.**
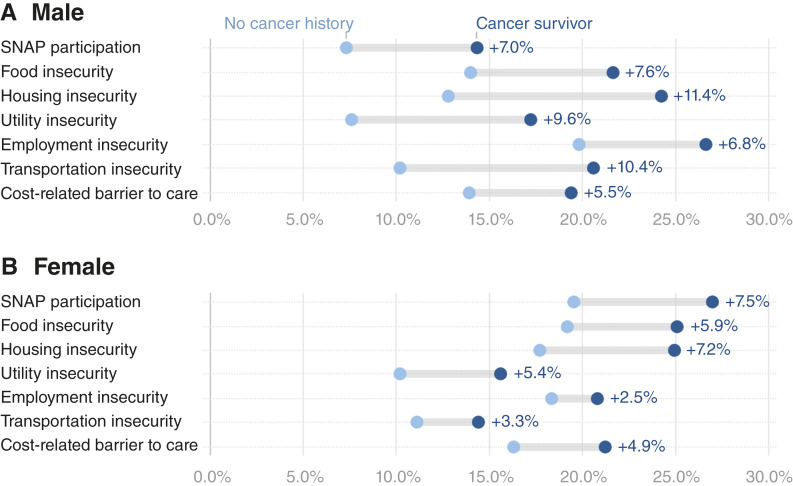
ADs in weighted prevalence (%) of individual social risks between cancer survivors versus adults without a cancer history, stratified by sex among young adults. ADs (cancer survivors minus adults without a cancer history) in prevalence of social risk factors among young adults (ages 18–39), stratified by race and ethnicity. **A,** Male. **B,** Female. Social risk factors include food insecurity (two items), housing insecurity, utility insecurity, employment insecurity, transportation insecurity, and cost-related barriers to healthcare. Estimates are weighted and derived from the 2022–2023 BRFSS.

ADs between young adults with and without a history of cancer were larger among males, except for SNAP participation in which the gap was comparable across sexes (AD = 7 vs. AD = 7.5; Fig. 3). Comparing young adults without a history of cancer, females consistently showed higher prevalence of social risks than males with the exception of employment insecurity. Among survivors, males had higher prevalence than females for utility, employment, and transportation insecurity, whereas female survivors had higher prevalence of SNAP participation, food insecurity, and cost-related barrier to care.

### Sensitivity analysis

Sensitivity analyses comparing participants from states deploying the optional SDHE module, those missing social risk data, and those included in the analytic sample revealed only modest differences in demographic and health characteristics (generally ≤3%), consistent with patterns typically associated with survey response (Supplementary Table S2). These findings suggest limited bias from differential nonresponse or item-level missingness. Notably, cancer history status did not differ across groups, suggesting minimal bias related to nonresponse or item-level missingness.

Consistent with descriptive findings, the prevalence of missing income increased with age, exceeding 90% among adults ≥65 years (Supplementary Table S3). Missingness was also substantial among middle-aged adults and present among younger adults, regardless of cancer history. In contrast, reported SNAP participation and cost-related barriers to care were relatively uncommon across all groups, even where income data were missing. These findings highlight the challenge of using income-based measures in population surveys and suggest that missingness may not fully align with other indicators of social vulnerability.

## Discussion

This nationally representative descriptive study examined the prevalence of seven individual-level social risks among adults with and without a history of cancer. Prior research has largely focused on medical financial hardship, often without including a cancer-free comparison group, leaving broader social vulnerabilities and life-course variation insufficiently studied. This study had two key findings. First, young adults (18–39 years) with a history of cancer reported higher prevalence of social risks compared with age-similar peers, whereas differences were attenuated in middle-aged adults and largely absent or reversed among older adults. Second, among young adults, patterns of disparity differed by state policy context and across demographic groups, reflecting structural inequities that influence social risk burden.

In age-stratified analyses, young adults exhibited the highest prevalence of social risks, with the largest ADs between survivors and those without a cancer history. This life stage is characterized by economic instability, transitional family and work roles, and limited access to social safety nets, leaving young adults particularly susceptible to financial strain. Housing and childcare costs, student debt, unstable employment, gaps in health insurance, and minimal employer-sponsored benefits compound these challenges, whereas cancer may further amplify burdens, forcing tradeoffs such as reducing spending on essentials or deferring long-term goals like retirement savings or education (27–31). Prior studies have similarly documented elevated financial and social hardship among young adult cancer survivors, and our findings extend this literature by demonstrating that these risks are further amplified relative to age-similar adults without cancer (4, 32, 33). These vulnerabilities may impede adherence to follow-up care, preventive screening, and engagement with supportive services, highlighting the urgent need to integrate systematic assessment and mitigation of social risks into survivorship care. In contrast, we found the lowest prevalence of social risks among older adults (≥65), and in some domains survivors seemed to fare better than those without cancer, likely reflecting the protective influence of Medicare insurance coverage, social security, and other age-based programs that stabilize access to healthcare and basic needs (34, 35). Collectively, these findings reinforce a growing body of literature among cancer survivors documenting pronounced age-related disparities in social risk and underscore the critical importance of life course–informed, age-sensitive interventions (9, 15, 36–38).

Within the high-risk young adult population, multiple overlapping social risks compounded vulnerability. Non–Medicaid expansion states exhibited larger differences between survivors and adults without a cancer history for SNAP participation, housing and utility insecurity, and cost-related barrier to care, with overall prevalence across all social risks higher in these states, consistent with evidence that Medicaid expansion can reduce financial strain, improve access to insurance coverage, and increase adherence to recommended medical care after cancer treatment (39–43). Racial and ethnic disparities were also evident. Although NH White young adults exhibited larger ADs between survivors and adults without a cancer history for most social risks due to lower prevalence among their peers, racially and ethnically minoritized groups faced persistently higher prevalence across all domains, reflecting structural inequities that elevate vulnerability regardless of cancer history (44–50). Sex differences revealed additional nuance. Males showed larger ADs across most social risks, whereas females without cancer consistently reported higher prevalence (except for employment insecurity). Among survivors, males experienced higher prevalence of utility, employment, and transportation insecurity than females, suggesting that cancer may differentially disrupt social resources depending on gendered roles and responsibilities (51–54). These findings illustrate baseline gender inequities in social risks among individuals without a history of cancer, with increased prevalence of social risks among women. The increased difference in social risks between male survivors and their peers may indicate social vulnerability in the face of a cancer diagnosis related to loss of employment and financial means related to gendered roles in the workforce (55). Moreover, high overall prevalence of social risks among women, even women without a history of cancer, reflects longstanding disparities in social determinants of health among women that may be further amplified after a cancer diagnosis (56, 57). Gender differences in employment and financial means may contribute to the sex differences in social risk burden noted among male and female cancer survivors. These patterns highlight the importance of considering both absolute and relative differences, as well as policy and demographic context, when evaluating disparities. Leveraging this evidence to prioritize support for young adult survivors (particularly those in non–Medicaid expansion states, racially and ethnically minoritized groups, and males) can help ensure that the highest-risk subgroups receive targeted resources and social support during survivorship.

### Limitations

This study has several limitations, primarily related to the use of self-reported, cross-sectional survey data. First, sample size constraints and data availability restricted the scope of analyses. Despite pooling 2 years of data, sparse cell sizes prevented reporting highly granular subgroup estimates (e.g., disaggregated racial and ethnic subgroups or narrower age bands). Similarly, although we used an optional BRFSS module to capture social risks, key survivorship-related variables (e.g., time since diagnosis, cancer type, and stage at diagnosis) as well as other social-context factors central to our study question (e.g., sexual orientation, gender identity, and perceived discrimination) were collected in different optional modules, administered by a limited subset of states; including multiple modules would have drastically reduced the analytic sample and state coverage. Future research should prioritize inclusion of these factors to better characterize heterogeneity in survivorship experiences. Second, self-reporting may introduce misclassification, particularly questions of cancer history (58, 59). However, BRFSS questions are generally reliable and validated, and self-report may outperform administrative data in some domains (e.g., race and ethnicity and perceived social risk; ref. 60). Third, despite the BRFSS’s complex sampling design and weighting (intended to mitigate selection bias), bias may persist as certain marginalized populations (e.g., institutionalized and unhoused populations) are not represented. In addition, the questionnaire structure may contribute to differential item nonresponse: optional modules follow the core questionnaire, and individuals reached by cell phone with out-of-state area codes only receive core items, which may exacerbate breakoff rates and exacerbate nonresponse bias (61, 62). Lastly, these findings reflect data from US states participating during 2022 to 2023 and capture a cross-sectional snapshot of social risks during that period. As a descriptive study, our goal was to characterize existing disparities rather than infer causality; the results should be interpreted within the context of the survey’s design and timeframe (19, 24).

Despite these limitations, this study offers several key strengths. First, it provides novel, nationally representative data on age-stratified social risks among cancer survivors, addressing a critical gap in the literature. Second, by examining seven individual-level social risk domains, we offer a more comprehensive assessment of vulnerabilities than prior studies, capturing the multidimensional nature of social disadvantage. Third, our analytic approach considered both absolute prevalence and ADs, allowing nuanced interpretation: whereas ADs highlight disparities relative to age-similar peers, high baseline prevalence in historically marginalized populations clarifies the additional burden attributable to cancer. Finally, the recency of the data provides a timely snapshot of social risks, supporting targeted screening, resource allocation, and interventions aimed at promoting equitable survivorship outcomes.

### Conclusion

This nationally representative study demonstrates that young adult cancer survivors experience the highest burden of multiple social risks, with disparities further shaped by state Medicaid expansion, race and ethnicity, and sex. By identifying subgroups most vulnerable during early adulthood, these findings extend understanding of social risk across the life course and underscore the influence of demographic and local context on survivorship vulnerabilities. Standardized approaches to social risk assessment may overlook this heterogeneity; integrating systematic evaluation with targeted, context-sensitive supports can mitigate inequities and enhance survivorship outcomes. Finally, disaggregating survivors by age is essential, highlighting young adult and working-age survivors as priority populations for both research and tailored interventions

## Supplementary Material

Figure S1 Flowchart of sample size selection.

Figure S2 Map of US states that deployed the BRFSS SDHE Module in (a) 2022, (b) 2023, and (c) Both Years Combined (Analytic Sample).

Figure S3 Absolute differences in social risk factor prevalence between cancer survivors and adults without a cancer history, stratified by health insurance status among young adults.

Supplementary Methods Additional information about the methods

Table S1 Definitions and Survey Items for Social Risks Included in the Analytic Sample, BRFSS 2022 and 2023.

Table S2 Sensitivity analysis of variable missingness: characteristics of included vs excluded participants.

Table S3 Sensitivity analysis: Patterns of missing household income by age, cancer history, food assistance, and cost-related care delays.

Table S4 Characteristics of study sample overall, and by age group.

Table S5 Social risks by age group.

Table S6 Number of social risks according to self-reported cancer history and by age group.

## Data Availability

The data that support the findings of this study are openly available from the US Centers for Disease Control and Prevention’s Behavioral Risk Factor Surveillance System at https://www.cdc.gov/brfss/index.html.
