# Supplementary material for: Social Risk Burden among US Cancer Survivors across Adulthood: Evidence from the 2022–2023 BRFSS
Source: Cancer Res Commun. 2026 Mar 16;6(3):566–76. doi: 10.1158/2767-9764.CRC-25-0664 (PMC13012017; doi:10.1158/2767-9764.CRC-25-0664)
Supplement: Figure S1 — Flowchart of sample size selection. [file crc-25-0664_figure_s1_suppsf1.pdf]

Figure S1. Flowchart of sample size selection.

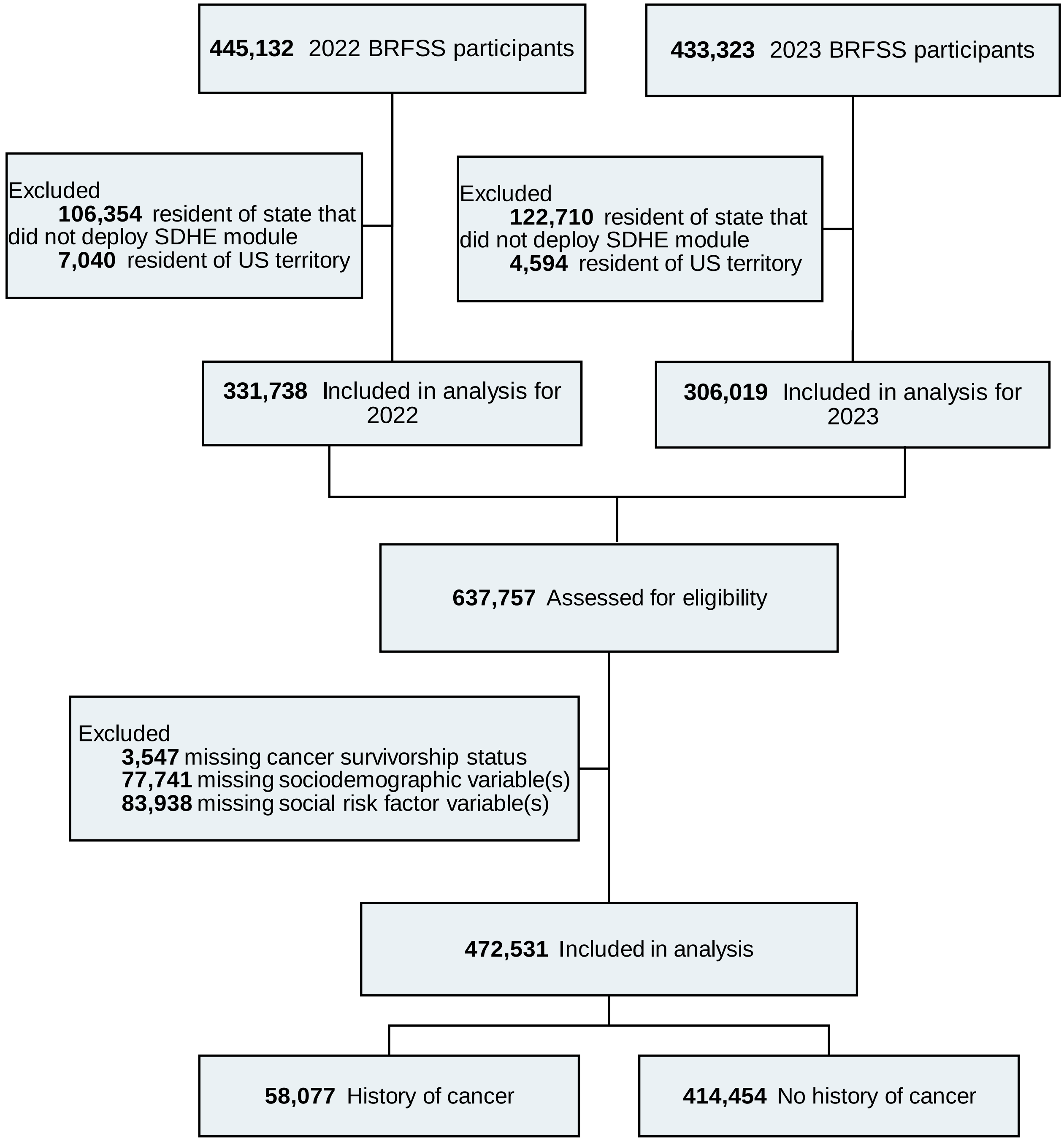

Abbreviations: BRFSS, Behavioral Risk Factor Surveillance System; SDoH/HE, Social Determinants of Health/Health Equity; no. is unweighted

[a]Demographic variables include: Educational attainment, Employment, FPL (number of adults, and/or refused number of children), home ownership, health insurance (except for don’t know/not sure), marital status, rural-urban status, veteran, disability, general health, Cigarette smoker, time since last routine checkup, personal doctor
