# Supplementary material for: Social Risk Burden among US Cancer Survivors across Adulthood: Evidence from the 2022–2023 BRFSS
Source: Cancer Res Commun. 2026 Mar 16;6(3):566–76. doi: 10.1158/2767-9764.CRC-25-0664 (PMC13012017; doi:10.1158/2767-9764.CRC-25-0664)

Figure S2. Map of US states that deployed the BRFSS SDHE Module in (a) 2022, (b) 2023, and (c) Both Years Combined (Analytic Sample).

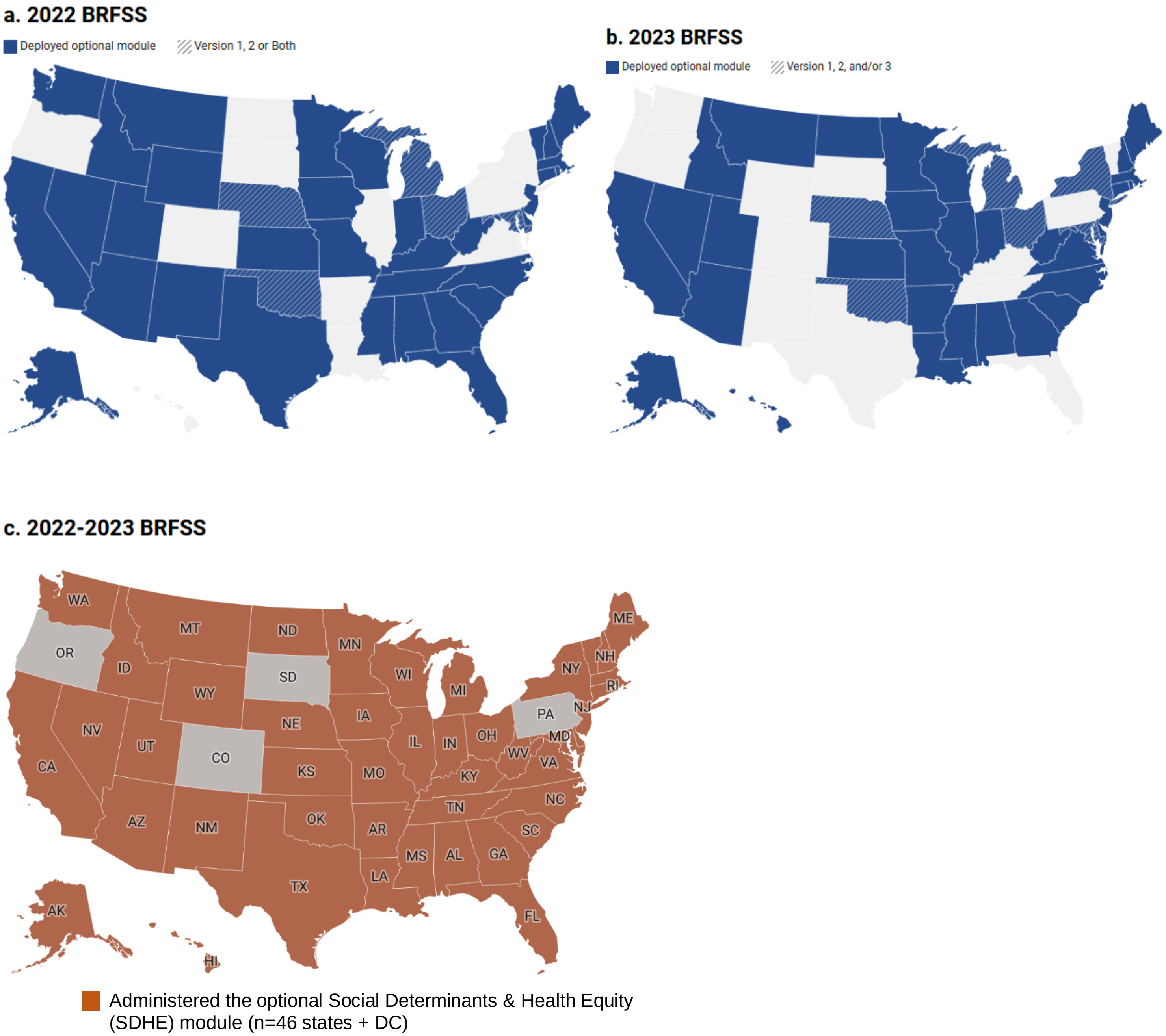

Supplement: Figure S2 — Map of US states that deployed the BRFSS SDHE Module in (a) 2022, (b) 2023, and (c) Both Years Combined (Analytic Sample). [file crc-25-0664_figure_s2_suppsf2.pdf]
