# Supplementary material for: Social Risk Burden among US Cancer Survivors across Adulthood: Evidence from the 2022–2023 BRFSS
Source: Cancer Res Commun. 2026 Mar 16;6(3):566–76. doi: 10.1158/2767-9764.CRC-25-0664 (PMC13012017; doi:10.1158/2767-9764.CRC-25-0664)
Supplement: Figure S3 — Absolute differences in social risk factor prevalence between cancer survivors and adults without a cancer history, stratified by health insurance status among young adults. [file crc-25-0664_figure_s3_suppsf3.pdf]

Figure S3. Absolute differences in social risk factor prevalence between cancer survivors and adults without a cancer history, stratified by health insurance status among young adults.

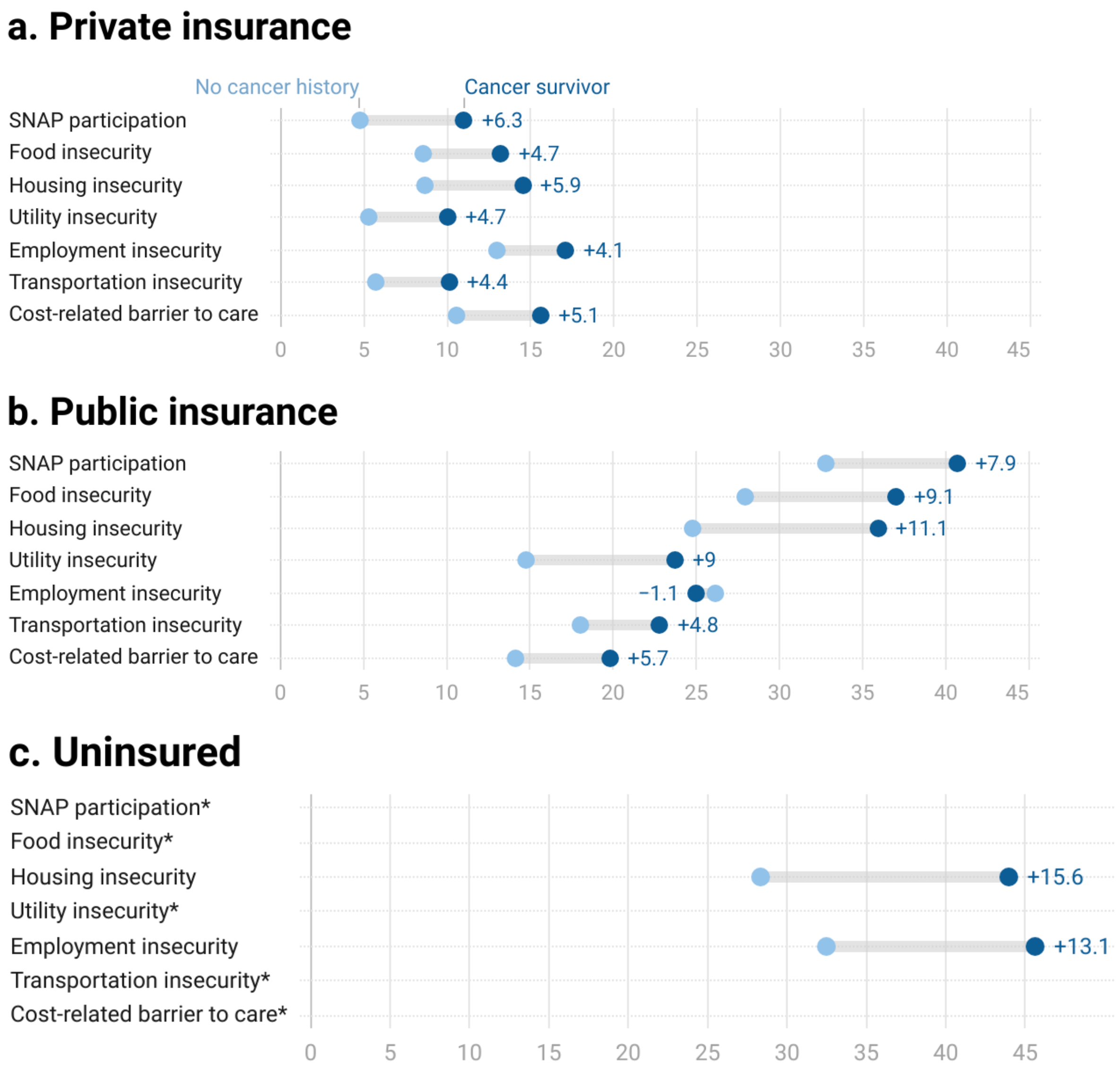

Absolute differences (cancer survivors minus adults without a cancer history) in prevalence of social risk factors among young adults (ages 18–39), stratified by insurance status. (a) private insurance. (b) public insurance. (c) uninsured. Social risk factors include food insecurity (two items), housing insecurity, utility insecurity, employment insecurity, transportation insecurity, and cost-related barriers to healthcare. Estimates are weighted and derived from the 2022–2023 Behavioral Risk Factor Surveillance System.

Asterisks (*) denotes data suppressed due to small unweighted sample sizes (<50)
