# Supplementary material for: Social Risk Burden among US Cancer Survivors across Adulthood: Evidence from the 2022–2023 BRFSS
Source: Cancer Res Commun. 2026 Mar 16;6(3):566–76. doi: 10.1158/2767-9764.CRC-25-0664 (PMC13012017; doi:10.1158/2767-9764.CRC-25-0664)
Supplement: Supplementary Methods — Additional information about the methods [file crc-25-0664_supplementary_methods_suppsm.docx]

**Supplementary Methods**

Data collection uses a dual-frame telephone methodology (landlines via disproportionate stratified sampling; cell phones via random sampling from the Telecordia database) with computer-assisted telephone interviewing (CATI). Survey data are weighted using iterative proportional fitting (raking) to account for differences between the sample and the target population.

The BRFSS median survey response rate for all states, territories, and DC in 2022, using the AAPOR R4 definition^1^, was 45.1% and ranged from 22.8% to 66.8%. Response rates for states included in this study sample had a median of 45.1% and ranged from 36.2% (California) to 59.6% (Alaska), For detailed information see the 2022 BRFSS Summary Data Quality Report.^2^

For 2023, the median survey response rate was 44.7% and ranged from 21.7% to 63.1%. During 2023, Kentucky and Pennsylvania were unable to collect enough data to meet the minimum requirements to be included in this public data set. Response rates for states included in this study sample had a median of 44.6% and ranged from 31.7% (Minnesota) to 63.1% (Alaska). For detailed information see the 2023 BRFSS Summary Data Quality Report.^3^ Despite relatively low response rates, BRFSS results are comparable to results from other national surveys.

Data cleaning procedures were applied to define the final analytic sample. Imputed BRFSS variables were used where applicable to reduce bias from missing data. Derived variables were created as needed, including collapsing sparse categories to improve statistical stability and confidentiality.

^1^The American Association for Public Opinion Research. Standard Definitions: Final Dispositions of Case Codes and Outcome Rates for Surveys (Revised 2023) Standards-Definitions-10th-edition.pdf (aapor.org) pp 86, 87.

^2^2022 Summary Data Quality Report. Centers for Disease Control and Prevention; 2023. Accessed September 3, 2025. https://www.cdc.gov/brfss/annual_data/2022/pdf/2022-DQR-508.pdf

^3^2023 Summary Data Quality Report. Centers for Disease Control and Prevention; 2023. Accessed September 3, 2025. https://www.cdc.gov/brfss/annual_data/2023/pdf/2022-DQR-508.pdf
