# Supplementary material for: Social Risk Burden among US Cancer Survivors across Adulthood: Evidence from the 2022–2023 BRFSS
Source: Cancer Res Commun. 2026 Mar 16;6(3):566–76. doi: 10.1158/2767-9764.CRC-25-0664 (PMC13012017; doi:10.1158/2767-9764.CRC-25-0664)
Supplement: Table S1 — Definitions and Survey Items for Social Risks Included in the Analytic Sample, BRFSS 2022 and 2023. [file crc-25-0664_table_s1_suppst1.docx]

**Table S1**. Definitions and Survey Items for Social Risks Included in the Analytic Sample, BRFSS 2022 and 2023.

| **Social risk factor** | **Question asked** | **Response options** | |
| --- | --- | --- | --- |
|  |  | **Score = 1** | **Score = 0** |
| **Employment Insecurity** | In the past 12 months have you lost employment or had hours reduced? | Yes | No |
| **SNAP participation** | During the past 12 months, have you received food stamps, also called SNAP, the Supplemental Nutrition Assistance Program on an EBT card? | Yes | No |
| **Food insecurity** | During the past 12 months how often did the food that you bought not last, and you didn’t have money to get more? Was that . . . | Always/usually/  sometimes | Never/rarely |
| **Housing insecurity** | During the last 12 months, was there a time when you were not able to pay your mortgage, rent or utility bills? | Yes | No |
| **Utility insecurity** | During the last 12 months was there a time when an electric, gas, oil, or water company threatened to shut off services? | Yes | No |
| **Transportation insecurity** | During the past 12 months has a lack of reliable transportation kept you from medical appointments, meetings, work, or from getting things needed for daily living? | Yes | No |
| **Cost-related barrier to care** | Was there a time in the past 12 months when you needed to see a doctor but could not because you could not afford it? | Yes | No |
