# Supplementary material for: Social Risk Burden among US Cancer Survivors across Adulthood: Evidence from the 2022–2023 BRFSS
Source: Cancer Res Commun. 2026 Mar 16;6(3):566–76. doi: 10.1158/2767-9764.CRC-25-0664 (PMC13012017; doi:10.1158/2767-9764.CRC-25-0664)
Supplement: Table S2 — Sensitivity analysis of variable missingness: characteristics of included vs excluded participants. [file crc-25-0664_table_s2_suppst2.docx]

**Table S2.** Sensitivity analysis of variable missingness: characteristics of included vs excluded participants.

|  | **Respondents, No. (Weighted %)** | | |
| --- | --- | --- | --- |
| **Characteristic** | **Administered the SDHE Module** | **Missing Social Risks (Excluded)** | **Final Study Sample** |
| **Total** | 637757 | 502115 | 472531 |
|  |  |  |  |
| **Sex** |  |  |  |
| Male | 300169 (48.6) | 232507 (47.8) | 218158 (47.5) |
| Female | 337588 (51.4) | 269608 (52.2) | 254373 (52.5) |
|  |  |  |  |
| **Age Group, y** |  |  |  |
| 18-39 | 144749 (36.8) | 108351 (34.9) | 100,628 (34.3) |
| 40-64 | 252290 (40.1) | 199042 (40.9) | 188,440 (41.3) |
| 65+ | 240718 (23.1) | 194722 (24.3) | 183,463 (24.4) |
|  |  |  |  |
| **Race and Ethnicity** |  |  |  |
| NH, White | 486872 (58.9) | 391186 (61.6) | 369603 (61.9) |
| NH, Black | 51674 (12.1) | 37714 (11.5) | 35170 (11.5) |
| NH, AI/AN | 10006 (1.2) | 7625 (1.2) | 7028 (1.2) |
| NH, AAPI | 18291 (6.7) | 13100 (6.1) | 12134 (6.0) |
| NH, Multiracial or Other | 15751 (3.5) | 12067 (3.4) | 11197 (3.3) |
| Hispanic | 55127 (17.5) | 40398 (16.2) | 37399 (16.0) |
| *Missing* | 36 (0.005) | 25 (0.003) | NA |
|  |  |  |  |
| **Educational Attainment** |  |  |  |
| Did not graduate HS | 36543 (11.6) | 25960 (10.7) | 23672 (10.4) |
| HS Diploma/GED | 156528 (27.2) | 119421 (26.8) | 111066 (26.4) |
| Some College | 170700 (30.1) | 135985 (30.8) | 128461 (31.0) |
| College Graduate | 270578 (30.5) | 219264 (31.4) | 209332 (32.2) |
| *Missing* | 3408 (0.6) | 1485 (0.3) | NA |
|  |  |  |  |
| **Employment Status** |  |  |  |
| Working | 317660 (55.8) | 253419 (56.9) | 241844 (57.9) |
| Out of Work (Looking) | 23629 (5.1) | 17859 (4.9) | 16564 (4.9) |
| Homemaker, Student or Retired | 244330 (29.9) | 198372 (31.1) | 187073 (31.2) |
| Unable to work | 38264 (6.3) | 29051 (6.2) | 27050 (6.1) |
| *Missing* | 13874 (2.9) | 3414 (0.9) | NA |
|  |  |  |  |
| **Marital/Partnership Status** |  |  |  |
| Married | 328619 (49.4) | 264981 (50.9) | 253361 (51.9) |
| Divorced/Separated/Widowed | 163962 (19.1) | 128993 (19.2) | 121566 (19.4) |
| Never Married | 138609 (30.3) | 104792 (29.1) | 97604 (28.7) |
| *Missing* | 6567 (1.2) | 3349 (0.7) | NA |
|  |  |  |  |
| **Insurance Type** |  |  |  |
| Commercial (Private) | 279275 (48.1) | 223223 (49.3) | 214586 (50.5) |
| Public | 299108 (38.9) | 238721 (39.4) | 227894 (40.0) |
| Uninsured | 32706 (7.8) | 23181 (7.0) | 21367 (6.8) |
| *Missing* | 26668 (5.3) | 6797 (1.4) | 8684 (2.7) |
|  |  |  |  |
| **Home Ownership** |  |  |  |
| Own | 449117 (66.7) | 364945 (69.7) | 347927 (71.0) |
| Rent | 151884 (25.6) | 111562 (23.4) | 104118 (23.2) |
| Other arrangement | 30369 (6.3) | 22435 (6) | 20486 (5.8) |
| *Missing* | 6387 (1.4) | 3173 (0.9) | NA |
|  |  |  |  |
| **Area of Residence** |  |  |  |
| Urban | 552454 (93.5) | 432692 (93.1) | 407240 (93.1) |
| Rural | 85095 (6.5) | 69249 (6.9) | 65291 (6.9) |
| *Missing* | 208 (0) | 174 (0) | NA |
|  |  |  |  |
| **US Census Region** |  |  |  |
| Northeast | 125398 (14.1) | 99862 (14.9) | 93557 (14.8) |
| Midwest | 179993 (23.7) | 144961 (25.5) | 137383 (25.8) |
| South | 175539 (36.4) | 130738 (34.4) | 122873 (34.3) |
| West | 156827 (25.8) | 126554 (25.2) | 118718 (25.1) |
|  |  |  |  |
| **Medicaid Expansion State** |  |  |  |
| No | 140313 (28.3) | 107897 (26.6) | 101473 (26.6) |
| Yes | 497444 (71.7) | 394218 (73.4) | 371058 (73.4) |
|  |  |  |  |
| **Veteran Status** |  |  |  |
| No | 555413 (89.0) | 441351 (90.3) | 415727 (90.4) |
| Yes | 76203 (9.7) | 59937 (9.5) | 56804 (9.6) |
| *Missing* | 6141 (1.3) | 827 (0.2) | NA |
|  |  |  |  |
| **Functional Disability Status** |  |  |  |
| No | 421844 (67.3) | 346821 (70.8) | 327392 (70.9) |
| Yes | 191598 (27.8) | 155279 (29.2) | 145139 (29.1) |
| *Missing* | 24315 (4.9) | 15 (0) | NA |
|  |  |  |  |
| **General Health Status** |  |  |  |
| Good or Better Health | 518768 (81.3) | 411342 (81.7) | 388735 (82.0) |
| Fair or Poor Health | 117183 (18.4) | 89803 (18.1) | 83796 (18.0) |
| *Missing* | 1806 (0.3) | 970 (0.2) | NA |
|  |  |  |  |
| **Cigarette Smoking Status** |  |  |  |
| Current Cigarette Use | 358868 (58.7) | 301949 (63.3) | 285414 (63.5) |
| Former Cigarette Use | 69083 (11.1) | 56457 (11.8) | 53267 (11.9) |
| Never Used Cigarettes | 165732 (22.0) | 140519 (24.3) | 133850 (24.7) |
|  | 44074 (8.2) | 3190 (0.6) | NA |
|  |  |  |  |
| **Has Personal Provider** |  |  |  |
| No | 79264 (17.0) | 57191 (15.3) | 52570 (14.9) |
| Yes | 552259 (81.7) | 441173 (83.8) | 419961 (85.1) |
| *Missing* | 6234 (1.3) | 3751 (0.9) | NA |
|  |  |  |  |
| **Time Since Checkup** |  |  |  |
| More than 1 year | 120296 (22.5) | 92442 (21.9) | 87489 (22.1) |
| 1 year or less | 509016 (75.8) | 404319 (76.7) | 385042 (77.9) |
| *Missing* | 8445 (1.6) | 5354 (1.3) | NA |
|  |  |  |  |
| **History of Cancer** |  |  |  |
| Yes | 74469 (8.0) | 60878 (8.5) | 58077 (8.7) |
| No | 559741 (91.5) | 438878 (91.1) | 414454 (91.3) |
| *Missing* | 3547 (0.5) | 2359 (0.4) | NA |

**Note**: missing includes, don’t know/not sure, refused, missing

Administered includes: Us states + DC (excluding territories)

only missing social risk factors (not CS history, or other covariates).
