# Supplementary material for: Social Risk Burden among US Cancer Survivors across Adulthood: Evidence from the 2022–2023 BRFSS
Source: Cancer Res Commun. 2026 Mar 16;6(3):566–76. doi: 10.1158/2767-9764.CRC-25-0664 (PMC13012017; doi:10.1158/2767-9764.CRC-25-0664)
Supplement: Table S3 — Sensitivity analysis: Patterns of missing household income by age, cancer history, food assistance, and cost-related care delays. [file crc-25-0664_table_s3_suppst3.docx]

**Table S3.** Sensitivity analysis: Patterns of missing household income by age, cancer history, food assistance, and cost-related care delays.

| **Age Group** | **Cancer History** | **% Missing Income** | **% Reporting SNAP participation** | **% Reporting cost-related barrier to care** |
| --- | --- | --- | --- | --- |
| **18–39** | **Yes** | 815 (50.9) | 344 (22.3) | 344 (20.5) |
| **18–39** | **No** | 57587 (58.8) | 11487 (13.5) | 14215 (15.1) |
| **40–64** | **Yes** | 12843 (76.8) | 1994 (12.7) | 1481 (10.9) |
| **40–64** | **No** | 119155 (66.0) | 17694 (11.1) | 15664 (10.1) |
| **65+** | **Yes** | 39257 (96.5) | 1902 (5.4) | 931 (2.5) |
| **65+** | **No** | 138184 (94.9) | 8449 (7.1) | 3746 (3.0) |
