# Supplementary material for: Social Risk Burden among US Cancer Survivors across Adulthood: Evidence from the 2022–2023 BRFSS
Source: Cancer Res Commun. 2026 Mar 16;6(3):566–76. doi: 10.1158/2767-9764.CRC-25-0664 (PMC13012017; doi:10.1158/2767-9764.CRC-25-0664)
Supplement: Table S4 — Characteristics of study sample overall, and by age group. [file crc-25-0664_table_s4_suppst4.docx]

**Table S4**. Characteristics of study sample overall, and by age group.

|  | **Total Sample** | **18–39** | **40–64 Years** | **≥65 years** |
| --- | --- | --- | --- | --- |
|  | (n=472,531) | (n=100,628 [34.3%]) | (n=188,440 [41.3%]) | (n=183,463 [24.4%]) |
| **Cancer History** |  |  |  |  |
| Cancer Survivor | 58077 (8.7) | 1,689 (1.5) | 16,063 (7.4) | 40,325 (20.9) |
| No History of Cancer | 414454 (91.3) | 98,939 (98.5) | 172,377 (92.6) | 143,138 (79.1) |
|  |  |  |  |  |
| **Sex** |  |  |  |  |
| Male | 218158 (47.5) | 51222 (49.2) | 86690 (47.8) | 80246 (44.5) |
| Female | 254373 (52.5) | 49406 (50.8) | 101750 (52.2) | 103217 (55.5) |
|  |  |  |  |  |
| **Race and Ethnicity** |  |  |  |  |
| Non-Hispanic American Indian or Alaska Native | 7028 (1.2) | 1807 (1.1) | 3362 (1.3) | 1859 (1.0) |
| Non-Hispanic Asian or Pacific Islander | 12134 (6.0) | 5329 (8) | 4673 (6.1) | 2132 (3.2) |
| Non-Hispanic Black | 35170 (11.5) | 8257 (11.8) | 15643 (12.3) | 11270 (9.6) |
| Non-Hispanic Multiracial | 11197 (3.3) | 3836 (4.2) | 4543 (3.2) | 2818 (2.3) |
| Non-Hispanic White | 369603 (61.9) | 64870 (52.1) | 144673 (61.3) | 160060 (76.9) |
| Hispanic | 37399 (16.0) | 16529 (22.8) | 15546 (15.7) | 5324 (7.0) |
|  |  |  |  |  |
| **Educational Attainment** |  |  |  |  |
| Did not graduate HS | 23672 (10.4) | 5704 (10.5) | 9868 (10.6) | 8100 (9.9) |
| HS Diploma/GED | 111066 (26.4) | 27195 (30.7) | 40047 (22.8) | 43824 (26.5) |
| Some College | 128461 (31.0) | 27140 (30.9) | 50103 (30.4) | 51218 (32) |
| College Graduate | 209332 (32.2) | 40589 (27.9) | 88422 (36.1) | 80321 (31.5) |
|  |  |  |  |  |
| **Employment Status** |  |  |  |  |
| Working | 241844 (57.9) | 77014 (71.5) | 134560 (70.8) | 30270 (16.8) |
| Out of Work^a^ | 16564 (4.9) | 6397 (7.5) | 8336 (4.8) | 1831 (1.2) |
| Homemaker, Student or Retired | 214123 (37.3) | 17217 (21.0) | 45544.0 (24.3) | 151362.0 (82.1) |
| Unable to work | 27050 (6.1) | 2646 (3.0) | 18092 (9.6) | 6312 (4.6) |
|  |  |  |  |  |
| **Marital/Partnership Status** |  |  |  |  |
| Married | 253361 (51.9) | 37656 (33.6) | 117306 (64) | 98399 (57.2) |
| Divorced/ Separated/ Widowed | 121566 (19.4) | 7072 (6.3) | 42709 (20.4) | 71785 (36.1) |
| Never Married | 97604 (28.7) | 55900 (60.1) | 28425 (15.6) | 13279 (6.8) |
|  |  |  |  |  |
| **Primary Health Insurance Coverage Type** |  |  |  |  |
| Commercial (Private) | 214586 (50.5) | 62173 (56.8) | 127163 (66.2) | 25250 (14.9) |
| Public | 227894 (40.0) | 24593 (26.9) | 48462 (25.7) | 154839 (82.8) |
| Medicare | 158064 (22.8) | 3590 (4.6) | 14827 (7.9) | 139647 (73.7) |
| Medicaid | 41737 (11.1) | 15081 (16.2) | 20785 (11.0) | 5871 (4.0) |
| Other | 28093 (6.2) | 5922 (6.0) | 12850 (6.9) | 9321 (5.1) |
| Uninsured | 21367 (6.8) | 9955 (11.4) | 10480 (6.6) | 932 (0.7) |
| Don’t Know/Not Sure | 8684 (2.7) | 3907 (4.9) | 2335 (1.4) | 2442 (1.6) |
|  |  |  |  |  |
| **Federal Poverty Level** |  |  |  |  |
| Under 138% | 24645 (8.2) | 11868 (13.7) | 10516 (7.2) | 2261 (2.0) |
| 139-250% | 22108 (6.0) | 10103 (9.7) | 10249 (5.8) | 1756 (1.2) |
| 137-400% | 25312 (6.4) | 10066 (8.8) | 14032 (7.7) | 1214 (0.8) |
| > 400% | 32625 (8.4) | 10189 (9.1) | 21645 (12.5) | 791 (0.6) |
| Missing^C^ | 367841 (71.0) | 58402 (58.7) | 131998 (66.8) | 177441 (95.4) |
|  |  |  |  |  |
| **Home Ownership** |  |  |  |  |
| Own | 347927 (71.0) | 43513 (47.3) | 145971 (80.1) | 158443 (88.7) |
| Rent | 104118 (23.2) | 45599 (39.7) | 37600 (17.7) | 20919 (9.4) |
| Other arrangement | 20486 (5.8) | 11516 (13.0) | 4869 (2.2) | 4101 (1.9) |
|  |  |  |  |  |
| **Area of residence** |  |  |  |  |
| Urban | 407240 (93.1) | 90298 (94.3) | 162883 (93.1) | 154059 (91.5) |
| Rural | 65291 (6.9) | 10330 (5.7) | 25557 (6.9) | 29404 (8.5) |
|  |  |  |  |  |
| **US Census Region** |  |  |  |  |
| Northeast | 93557 (14.8) | 18739 (14.6) | 37989 (14.9) | 36829 (14.9) |
| Midwest | 137383 (25.8) | 29881 (26.1) | 55203 (25.3) | 52299 (26.2) |
| South | 122873 (34.3) | 24673 (33.4) | 49114 (34.8) | 49086 (34.7) |
| West | 118718 (25.1) | 27335 (25.9) | 46134 (25.0) | 45249 (24.2) |
|  |  |  |  |  |
| **Medicaid Expansion State** |  |  |  |  |
| No | 101473 (26.6) | 20062 (25.7) | 40132 (27.1) | 41279 (27.1) |
| Yes | 371058 (73.4) | 80566 (74.3) | 148308 (72.9) | 142184 (72.9) |
|  |  |  |  |  |
| **Veteran Status** |  |  |  |  |
| No | 415727 (90.4) | 94961 (94.8) | 170804 (91.3) | 149962 (82.5) |
| Yes | 56804 (9.6) | 5667 (5.2) | 17636 (8.7) | 33501 (17.5) |
|  |  |  |  |  |
| **Functional Disability Status** |  |  |  |  |
| No | 327392 (70.9) | 78173 (76.2) | 139705 (73.8) | 109514 (58.5) |
| Yes | 145139 (29.1) | 22455 (23.8) | 48735 (26.2) | 73949 (41.5) |
|  |  |  |  |  |
| **General Health Status** |  |  |  |  |
| Good or Better Health | 388735 (82.0) | 88735 (87.2) | 154738 (80.8) | 145262 (76.6) |
| Fair or Poor Health | 83796 (18.0) | 11893 (12.8) | 33702 (19.2) | 38201 (23.4) |
|  |  |  |  |  |
| **Cigarette Use Status** |  |  |  |  |
| Never Smoked | 285414 (63.5) | 72994 (74.3) | 111503 (59.8) | 100917 (54.5) |
| Current Smoker | 53267 (11.9) | 11263 (11.0) | 27886 (14.7) | 14118 (8.3) |
| Former Smoker | 133850 (24.7) | 16371 (14.7) | 49051 (25.5) | 68428 (37.2) |
|  |  |  |  |  |
| **Personal Healthcare Provider** |  |  |  |  |
| No | 52570 (14.9) | 25879 (27) | 19786 (11.4) | 6905 (3.8) |
| Yes | 419961 (85.1) | 74749 (73) | 168654 (88.6) | 176558 (96.2) |
|  |  |  |  |  |
| **Time Since Routine Checkup** |  |  |  |  |
| More than 1 year | 87489 (22.1) | 34879 (34.2) | 37539 (20.4) | 15071 (7.8) |
| 1 year or less | 385042 (77.9) | 65749 (65.8) | 150901 (79.6) | 168392 (92.2) |
|  |  |  |  |  |
| **Survey Year** |  |  |  |  |
| 2022 | 243104 (50.3) | 52000 (50.3) | 98800 (50.6) | 92304 (49.9) |
| 2023 | 229427 (49.7) | 48628 (49.7) | 89640 (49.4) | 91159 (50.1) |

**Abbreviations**: Wt, weighted; GED, general education diploma; NA, not applicable.

Percentages represent weighted column percentages.

Percentages may not add to 100% due to rounding.
