# Supplementary material for: Social Risk Burden among US Cancer Survivors across Adulthood: Evidence from the 2022–2023 BRFSS
Source: Cancer Res Commun. 2026 Mar 16;6(3):566–76. doi: 10.1158/2767-9764.CRC-25-0664 (PMC13012017; doi:10.1158/2767-9764.CRC-25-0664)
Supplement: Table S5 — Social risks by age group. [file crc-25-0664_table_s5_suppst5.docx]

**Table S5**. Social risks by age group.

| **Social Risk Factors** | **Total Sample** | **Age Group** | | |
| --- | --- | --- | --- | --- |
|  |  | **18–39 Years** | **40–64 Years** | **≥65 years** |
|  | (n=472,531) | (n=100,628) | (n=188,440) | (n=183,463) |
| **SNAP Participation** |  |  |  |  |
| No | 430661 (89.1) | 88797 (86.4) | 168752 (88.8) | 173112 (93.2) |
| Yes | 41870 (10.9) | 11831 (13.6) | 19688 (11.2) | 10351 (6.8) |
| **Food Insecurity** |  |  |  |  |
| No | 423669 (86.4) | 85980 (83.3) | 166156 (86.2) | 171533 (91.4) |
| Yes | 48862 (13.6) | 14648 (16.7) | 22284 (13.8) | 11930 (8.6) |
| **House Insecurity** |  |  |  |  |
| No | 432040 (88.6) | 86383 (84.6) | 168095 (87.9) | 177562 (95.6) |
| Yes | 40491 (11.4) | 14245 (15.4) | 20345 (12.1) | 5901 (4.4) |
| **Utility Insecurity** |  |  |  |  |
| No | 444996 (92.7) | 91991 (91.0) | 173817 (91.6) | 179188 (97.1) |
| Yes | 27535 (7.3) | 8637 (9.0) | 14623 (8.4) | 4275 (2.9) |
| **Employment Insecurity** |  |  |  |  |
| No | 430881 (87.8) | 83644 (80.9) | 168898 (88.1) | 178339 (96.9) |
| Yes | 41650 (12.2) | 16984 (19.1) | 19542 (11.9) | 5124 (3.1) |
| **Transportation Insecurity** |  |  |  |  |
| No | 444226 (92.5) | 91003 (89.3) | 176208 (93.2) | 177015 (96.1) |
| Yes | 28305 (7.5) | 9625 (10.7) | 12232 (6.8) | 6448 (3.9) |
| **Cost-related barrier to care** |  |  |  |  |
| No | 436150 (89.9) | 86069 (84.8) | 171295 (89.8) | 178786 (97.1) |
| Yes | 36381 (10.1) | 14559 (15.2) | 17145 (10.2) | 4677 (2.9) |
|  |  |  |  |  |
| **Cumulative Social Risk, Mean (±STD)** | 0.73 (0.004) | 1.0 (0.01) | 0.74 (0.01) | 0.33 (0.005) |

**Note**: Cumulative Social Risk ranged from 0-7

**Abbreviations**: SNAP, Supplemental Nutrition Assistance Program (food stamps); STD, Standard deviation.
