# Supplementary material for: Social Risk Burden among US Cancer Survivors across Adulthood: Evidence from the 2022–2023 BRFSS
Source: Cancer Res Commun. 2026 Mar 16;6(3):566–76. doi: 10.1158/2767-9764.CRC-25-0664 (PMC13012017; doi:10.1158/2767-9764.CRC-25-0664)
Supplement: Table S6 — Number of social risks according to self-reported cancer history and by age group. [file crc-25-0664_table_s6_suppst6.docx]

**Table S6.** Number of social risks according to self-reported cancer history and by age group.

|  | **No. Social Risks** | **Total Sample** | **Cancer Survivors** | **No History of Cancer** |
| --- | --- | --- | --- | --- |
| **Total Sample** | **0** | 340850 (65.5) | 45790 (74.5) | 295060 (64.6) |
|  | **1** | 66868 (16.6) | 6712 (13.1) | 60156 (16.9) |
|  | **2** | 28396 (7.5) | 2479 (5.0) | 25917 (7.7) |
|  | **3+** | 36417 (10.5) | 3096 (7.5) | 33321 (10.8) |
| **18–39** | **0** | 58505 (54.4) | 807 (45.9) | 57698 (54.5) |
|  | **1** | 19456 (20.8) | 308 (19.3) | 19148 (20.8) |
|  | **2** | 9384 (10.1) | 176 (10.3) | 9208 (10.1) |
|  | **3+** | 13283 (14.8) | 398 (24.5) | 12885 (14.6) |
| **40–64 Years** | **0** | 129126 (65.7) | 10658 (64.7) | 118468 (65.8) |
|  | **1** | 28078 (15.9) | 2458 (1.06) | 25620 (15.9) |
|  | **2** | 12863 (7.3) | 1144 (6.9) | 11719 (7.3) |
|  | **3+** | 18373 (11.1) | 1803 (12.4) | 16570 (11.0) |
| **65+ Years** | **0** | 153219 (80.6) | 34325 (83.1) | 118894 (79.9) |
|  | **1** | 19334 (11.8) | 3946 (10.7) | 15388 (12.1) |
|  | **2** | 6149 (4.2) | 1159 (3.3) | 4990 (4.4) |
|  | **3+** | 4761 (3.5) | 895 (2.9) | 3866 (3.6) |
